# Pregnancy and lactation interfere with the response of autoimmunity to modulation of gut microbiota

**DOI:** 10.1186/s40168-019-0720-8

**Published:** 2019-07-16

**Authors:** Qinghui Mu, Xavier Cabana-Puig, Jiangdi Mao, Brianna Swartwout, Leila Abdelhamid, Thomas E. Cecere, Haifeng Wang, Christopher M. Reilly, Xin M. Luo

**Affiliations:** 1 Department of Biomedical Sciences and Pathobiology, College of Veterinary Medicine, Virginia Tech, Blacksburg, VA USA; 2 Translational Biology, Medicine, and Health Graduate Program, Virginia Tech Carilion Research Institute, Virginia Tech, Roanoke, VA USA; 3 College of Animal Science, Key Laboratory of Molecular Animal Nutrition, Zhejiang University, Hangzhou, Zhejiang People’s Republic of China; 4 Edward Via College of Osteopathic Medicine, Blacksburg, VA USA

**Keywords:** Lupus, Pregnancy, Gut microbiota, *Lactobacillus animalis*, IDO, Treg, IFNγ, Leaky gut

## Abstract

**Background:**

Dysbiosis of gut microbiota exists in the pathogenesis of many autoimmune diseases, including systemic lupus erythematosus (lupus). Lupus patients who experienced pregnancy usually had more severe disease flares post-delivery. However, the possible role of gut microbiota in the link between pregnancy and exacerbation of lupus remains to be explored.

**Results:**

In the classical lupus mouse model MRL/lpr, we compared the structures of gut microbiota in pregnant and lactating individuals vs. age-matched naïve mice. Consistent with studies on non-lupus mice, both pregnancy and lactation significantly changed the composition and diversity of gut microbiota. Strikingly, modulation of gut microbiota using the same strategy resulted in different disease outcomes in postpartum (abbreviated as “PP,” meaning that the mice had undergone pregnancy and lactation) vs*.* control (naïve; i.e., without pregnancy or lactation) MRL/lpr females; while vancomycin treatment attenuated lupus in naïve mice, it did not do so, or even exacerbated lupus, in PP mice. *Lactobacillus animalis* flourished in the gut upon vancomycin treatment, and direct administration of *L. animalis* via oral gavage recapitulated the differential effects of vancomycin in PP vs. control mice. An enzyme called indoleamine 2,3-dioxygenase was significantly inhibited by *L. animalis*; however, this inhibition was only apparent in PP mice, which explained, at least partially, the lack of beneficial response to vancomycin in these mice. The differential production of immunosuppressive IL-10 and proinflammatory IFNγ in PP vs*.* control mice further explained why the disease phenotypes varied between the two types of mice bearing the same gut microbiota remodeling strategy.

**Conclusions:**

These results suggest that pregnancy and lactation interfere with the response of autoimmunity to modulation of gut microbiota. Further studies are necessary to better understand the complex relationship between pregnancy and lupus.

**Electronic supplementary material:**

The online version of this article (10.1186/s40168-019-0720-8) contains supplementary material, which is available to authorized users.

## Background

Autoimmune diseases occur when individuals have disturbances in immune responses, especially T and B cell responses, which in turn attack self-antigens and subsequently cause tissue damage [1]. Comprised of more than 70 different disorders, autoimmune diseases affect more than 7% of the general population [2]. Many of these diseases are more prevalent in females [3]. Sex hormones contribute to this phenomenon by regulating the immune system via hormone receptors [4]. During pregnancy, to support fetal development, the maternal body undergoes changes on hormones, immunity, and metabolism [5]. Evidences suggest that there is an association between pregnancy and autoimmune diseases [3, 6]. However, except for hormonal effects, it remains unclear how pregnancy influences the autoimmunity [7].

Systemic lupus erythematosus, or lupus, is a prototypical systemic autoimmune disease characterized by severe and persistent multiorgan inflammation [8]. The loss of self-tolerance and activation of autoreactive immune cells lead to the production of autoantibodies and diverse pro-inflammatory cytokines, pathogenic cell infiltration, and subsequent tissue damage in multiple organs, including, but not limited to, the skin, kidney, lung, heart, and brain [9]. With more than half of patients affected, kidney inflammation, or lupus nephritis, is the leading cause of lupus mortality [10]. Although affecting both females and males, lupus is a highly gender-biased disease with about 9 times more incidences in females of child-bearing age. Pregnant women with lupus have a higher risk of lupus flares post-delivery [11]. Therefore, some clinicians recommend women with lupus to avoid pregnancy [12–15]. A better understanding of the mechanisms underlying pregnancy-induced disease flares and the development of new therapeutic strategies for pregnant women will likely benefit these patients for successful pregnancies and better lupus outcomes.

The exact mechanism of pathogenesis is still unclear for lupus, but genetics, hormones, and environmental factors have been suggested as the major causes [8]. As an important factor linking environmental factors with the host, gut microbiota and its interaction with host health and disease have drawn a lot of attention in the past decade [16]. Increasing evidences indicate that a perturbed gut microbiota is associated with multiple autoimmune disorders such as lupus [17]. On the one hand, dysbiosis of gut microbiota does exist in both human patients and lupus-prone mouse models [18–20]. On the other hand, remodeling of gut microbiota community by antibiotics and probiotics has been shown to efficiently modulate disease development in lupus-prone mice [21–23]. These evidences have demonstrated the involvement of gut microbiota in lupus pathogenesis and suggested modulating gut microbes as a potential therapeutic method. Three potential mechanisms may explain the role of gut microbiota in lupus: (1) a leaky gut and associated bacterial translocation [23–25], (2) molecular mimicry [26], and (3) induction of regulatory immune response [22]. But additional mechanisms may exist. For example, the capability of gut microbiota to interact with host hormones and molecules produced by microbes, such as short chain fatty acids (SCFAs), can potentially influence lupus pathogenesis [27, 28]. Furthermore, despite a higher risk of lupus flares in pregnant patients post-delivery [11, 15], the role of gut microbiota in the link between pregnancy and exacerbated lupus has never been examined.

The current study is driven by an interesting finding—that vancomycin treatment attenuated lupus nephritis in naïve MRL/lpr females [21] but somehow exacerbated the disease in MRL/lpr mice that had experienced pregnancy and lactation (named postpartum or “PP” mice). In contrast to downregulating IL-6 and IL-17 production in control or naïve mice, the oral antibiotic was not able to affect either IL-6 or IL-17 expression in PP mice. Instead, in PP mice, oral vancomycin administration resulted in reduced regulatory T (Treg) and IL-10 producing B cell (B10) responses and subsequently a dampened anti-inflammatory IL-10 response. At the same time, the production of proinflammatory IFNγ was elevated. Importantly, the imbalance of IL-10 and IFNγ occurred only in PP but not naïve MRL/lpr mice. This provides an explanation of the differed disease manifestations between these two types of mice receiving the same vancomycin treatment. As pregnancy and lactation can significantly change the gut microbiota diversity and composition [29], and that vancomycin is not absorbed in gastrointestinal tract and thus not systemic [30], we analyzed the local gut microbiota status. As anticipated, vancomycin removed the majority of bacteria and greatly increased the relative abundance of *Lactobacillus* spp. [31, 32], particularly the species *Lactobacillus animalis*. We next determined whether weekly *L. animalis* oral gavage would recapitulate the differential effects of vancomycin on lupus disease in control vs. PP MRL/lpr mice. Indeed, direct administration of *L. animalis* significantly worsened lupus disease in PP mice. Consistently, *L. animalis* also decreased IL-10 but increased IFNγ. Further investigations showed that an enzyme indoleamine 2,3-dioxygenase (IDO) was inhibited by the enriched *L. animalis*; however, this inhibition was only apparent in PP mice. As IDO is known to be able to activate Treg cells [33–35], this observation may be able to explain the differential effects of vancomycin in control vs. PP mice. Together, these results provide a potential mechanism by which pregnancy and lactation may interfere with the response of autoimmunity to modulation of gut microbiota.

## Results

### Lack of beneficial response to oral vancomycin in PP MRL/lpr mice

**Fig. 1:**
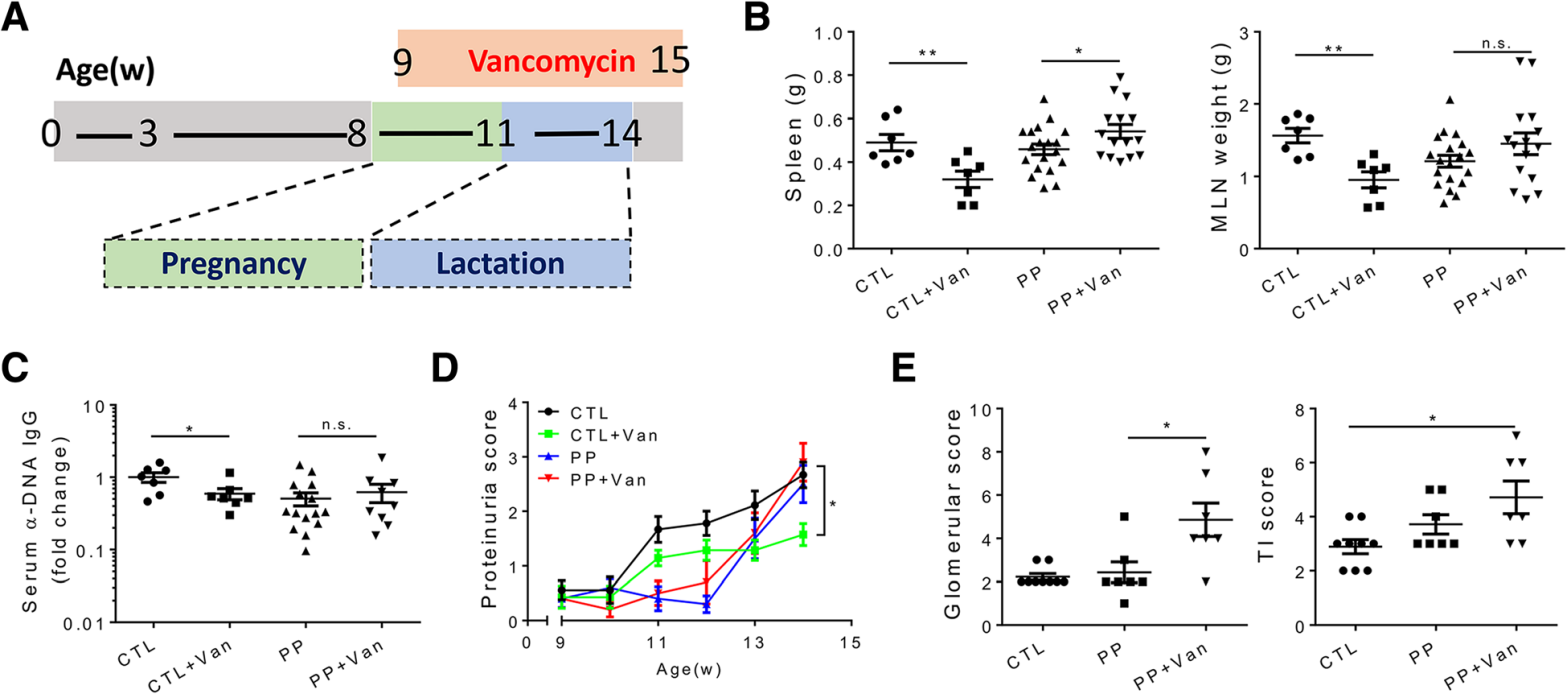
Lack of beneficial response to vancomycin in PP MRL/lpr mice. **a** Study design. PP mice were mated at 8 weeks of age, and only those delivered at 11 weeks old (± 1 day) were used in this study. Oral vancomycin was initiated at 9 weeks of age and ended at 15 weeks (endpoint). **b** Spleen and mesenteric lymph node (MLN) weights at 15 weeks of age. **c** Relative level of anti-DNA IgG in the mouse serum at 15 weeks of age. **d** Level of proteinuria over time. **e** Renal glomerular and tubulointerstitial (TI) scores. In **b**–**e**, *n* ≥ 7 in each group. *p < 0.05, **p < 0.01, n.s. not statistically significant

In our previous study, lupus disease was greatly attenuated in female MRL/lpr mice receiving oral vancomycin from 9 weeks to 15 weeks of age (the endpoint) [21]. However, when the exactly same treatment procedure was implemented on PP MRL/lpr females (Fig. 1a), the beneficial effect was not observed (Fig. 1b–e). As reported previously, vancomycin administration significantly decreased splenomegaly and the size of mesenteric lymph nodes (MLN) in age-matched naïve MRL/lpr mice (labeled “CTL”). On the contrary, the antibiotic treatment worsened splenomegaly in PP mice (Fig. 1b). The level of anti-DNA antibodies, a hallmark of disease in both lupus patients and lupus-prone animal models [8, 36], was significantly reduced by vancomycin in naïve mice but not PP mice (Fig. 1c and Additional file 1: Figure S1A). Notably, the level of this autoantibody was even increased in vancomycin-treated PP mice when they were analyzed at 11 weeks of age and at the time of delivery (Additional file 1: Figure S1B). To assess kidney inflammation and function, we measured the level of proteinuria weekly and had the kidney sliced and scored by a pathologist. In control mice, the proteinuria level was significantly decreased by vancomycin treatment (Fig. 1d). Correspondingly, the glomerular and tubulointerstitial (TI) scores were both lowered (data not shown and [21]). However, in PP mice, the proteinuria level was not influenced by vancomycin (Fig. 1d). Interestingly, the level of proteinuria decreased around the time pups were delivered and quickly caught up afterwards both with and without vancomycin treatment. Renal injury was more severe in vancomycin-treated PP mice as suggested by significantly higher kidney histopathological scores (Fig. 1e). Taken together, these data indicate that contrary to the protective effect on naïve mice, vancomycin treatment was not beneficial but detrimental in MRL/lpr mice that had experienced pregnancy and lactation.

### Vancomycin-mediated downregulation of regulatory immune response in PP MRL/lpr mice

**Fig. 2:**
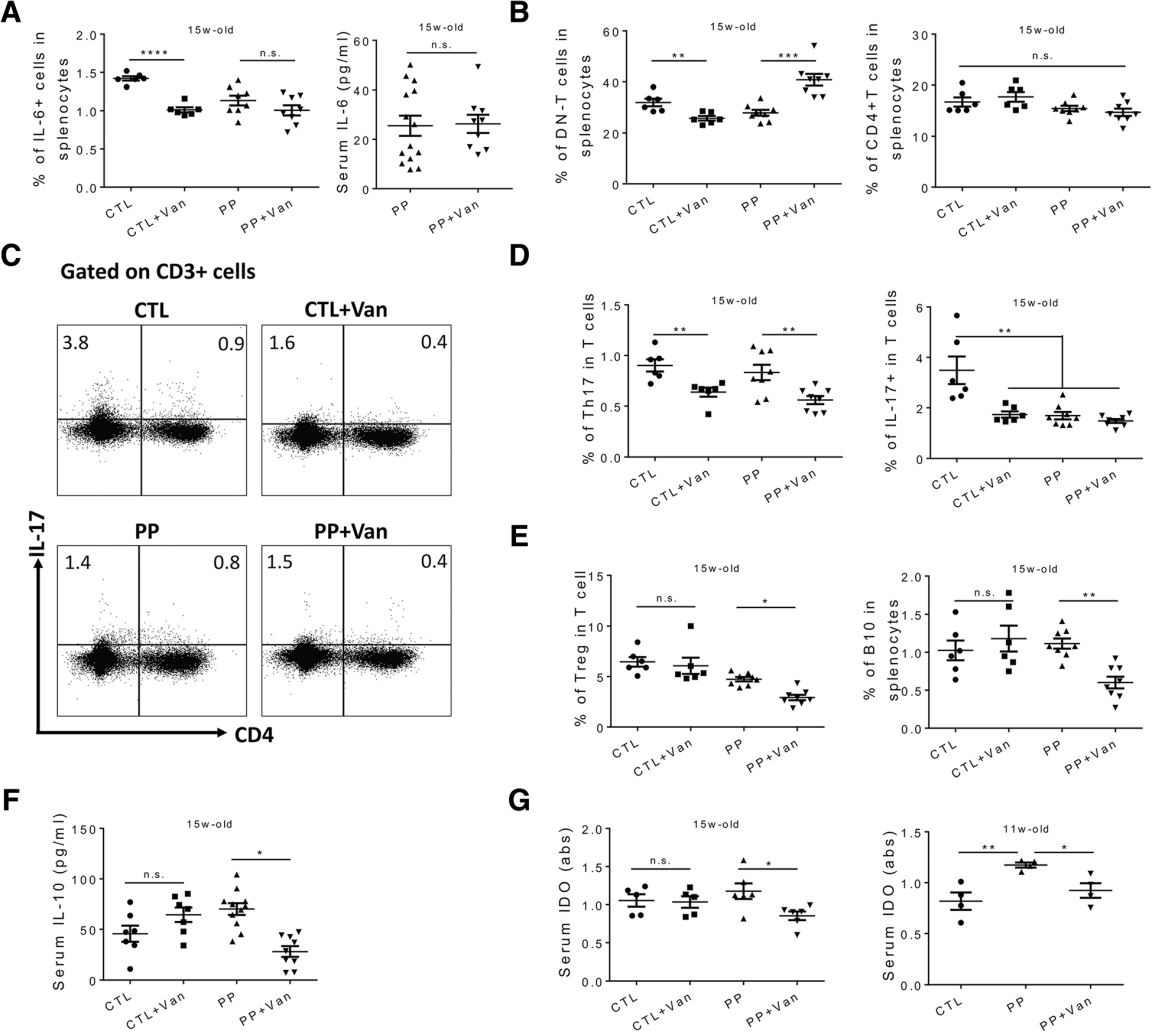
Vancomycin-mediated downregulation of regulatory immune response in PP MRL/lpr mice. **a** Left: percentage of IL-6 producing cells in the spleen. Right: serum level of IL-6. Both were at 15 weeks of age. **b** Percentage of DN-T cell (CD3^+^CD4^−^CD8^−^) and CD4^+^ T cells (CD3^+^CD4^+^CD8^−^) in the spleen at the 15 weeks of age (*n* ≥ 6 per group). **c** Representative FACS plots of IL-17 producing T cells. **d** Percentage of Th17 cells and IL-17 producing T cells (CD3^+^IL-17^+^) in the spleen at the 15 weeks of age (*n* ≥ 6 per group). **e** Percentage of Treg (CD3^+^CD4^+^Foxp3^+^) and B10 (CD19^+^IL-10^+^) cells in the spleen at the 15 weeks of age (*n* ≥ 6 per group). **f** Level of IL-10 in mouse serum at the 15 weeks of age (*n* ≥ 7 per group). **g** Level of IDO in mouse serum at the 15 weeks and 11 weeks of age (*n* ≥ 4 per group). *p < 0.05, **p < 0.01, ***p < 0.001, ****p < 0.0001, n.s. not statistically significant

We next sought to explore the underlying mechanism(s) behind the phenomenon that the same antibiotic treatment led to opposite disease outcomes in control *vs.* PP mice. We examined different immune cell populations and diverse inflammatory mediators, especially those related to IL-17 (Additional file 1: Figure S2A), as the production of this cytokine was ameliorated in naïve mice receiving vancomycin [21]. IL-6 is known as an important pathogenic cytokine in both human and mouse lupus due to, at least partially, its ability to induce Th17 cell differentiation and IL-17 production [37, 38]. In age-matched control mice, oral vancomycin significantly reduced IL-6 level and subsequently resulted in lower IL-17 production from different cellular resources (Fig. 2a–d and [21]). In contrast, the circulating IL-6 in PP mice remained unchanged after vancomycin treatment (Fig. 2a). In addition, although CD4^+^ and CD8^+^ T cells were not affected (Fig. 2b and Addditional file 1: Figure S2B), the percentage of double negative T (DN-T) cells in the spleen was changed by vancomycin in both control and PP mice, but in an opposite fashion (Fig. 2b). In both lupus patients and mouse models, DN-T cells expand and emerge as a major IL-17 producer [39, 40]. Remarkably, in control mice, vancomycin treatment not only reduced the percentage of DN-T cells but also reduced the proportion of DN-T cell capable of secreting IL-17 (Additional file 1: Figure S2C). It was a different story for PP MRL/lpr mice. Pregnancy and lactation significantly increased the percentage of DN-T cells (Fig. 2b), but it appears that the experiences dampened the capability of DN-T cells to secrete IL-17, which was not further changed by vancomycin (Additional file 1: Figure S2C). Apart from DN-T cells, we also investigated Th17 cells which secrete IL-17 as the signature cytokine [41]. Interestingly, oral vancomycin leads to a similar decrease of Th17 cells in both control and PP mice (Fig. 2d). Considering the differed DN-T and Th17 responses in PP mice upon vancomycin treatment, we analyzed the total IL-17 expressing T cells (Fig. 2c). While vancomycin significantly downregulated the percentage of IL-17 expressing T cells in control mice, PP mice exhibited similar IL-17 producing abilities among all T cells with or without vancomycin (Fig. 2d). It is noteworthy that these changes were evident in the spleen, but not in the mesenteric lymph node (MLN; data not shown). Taken together, these results indicate that vancomycin treatment led to dampened IL-6 and IL-17 production and attenuated lupus nephritis in control mice but failed to change either the IL-17 response or disease manifestations in PP mice.

Opposing the pro-inflammatory function of IL-17 in autoimmune lupus, Treg cells play a central regulatory role in suppressing disease development and progression [42, 43]. We thus measured splenic CD4^+^Foxp3^+^ Treg cells (Additional file 1: Figure S2A) and found that the vancomycin treatment resulted in a significant decrease of Treg cells in PP but not control mice (Fig. 2e). We also assessed the percentage of CD19^+^IL-10^+^ cells in the spleen, which are B10 cells that have emerged as an important cell type regulating diverse autoimmune disorders [44]. A similar decrease of B10 cells was noted in PP but not control mice (Fig. 2e). As a result, the serum IL-10 concentration was significantly reduced in PP mice receiving vancomycin, but remained unchanged in control mice (Fig. 2f). As both regulatory B (Breg) cells and Treg cells can secrete IL-35, another important anti-inflammatory cytokine [45], we measured the serum level of IL-35. Surprisingly, IL-35 was not changed with or without vancomycin treatment (Additional file 1: Figure S2D), which was not consistent with the observed downregulation of Treg and B10 cells. It suggests that other IL-35 producing cells may exist in MRL/lpr mice [46].

Next, we set to evaluate the factor(s) contributing to the differential outcomes of regulatory cells and IL-10 production in control vs*.* PP mice. During pregnancy, the maternal immune system is dampened to tolerate and accept the fetus [47]. Different mechanisms are involved in pregnancy-induced immune tolerance, and IDO is one of them [48]. IDO is capable of promoting Treg differentiation and therefore plays a role in regulating autoimmune diseases [33, 34]. At 11 weeks of age (the time of delivery), PP MRL/lpr females had significantly higher IDO expression compared to age-matched naïve mice (Fig. 2g), although the increase of IDO was not apparent at 15 weeks of age when the pups had been weaned. Consistent with the observed changes for Treg cells (Fig. 2e) and serum IL-10 (Fig. 2f), vancomycin treatment significantly reduced the serum level of IDO only in PP mice (Fig. 2g). This observation provides a possible explanation for the differential effects of vancomycin on lupus disease in control vs*.* PP mice.

### Identification of a bacterium responsible for the differential effects of vancomycin on control vs. PP MRL/lpr mice

**Fig. 3:**
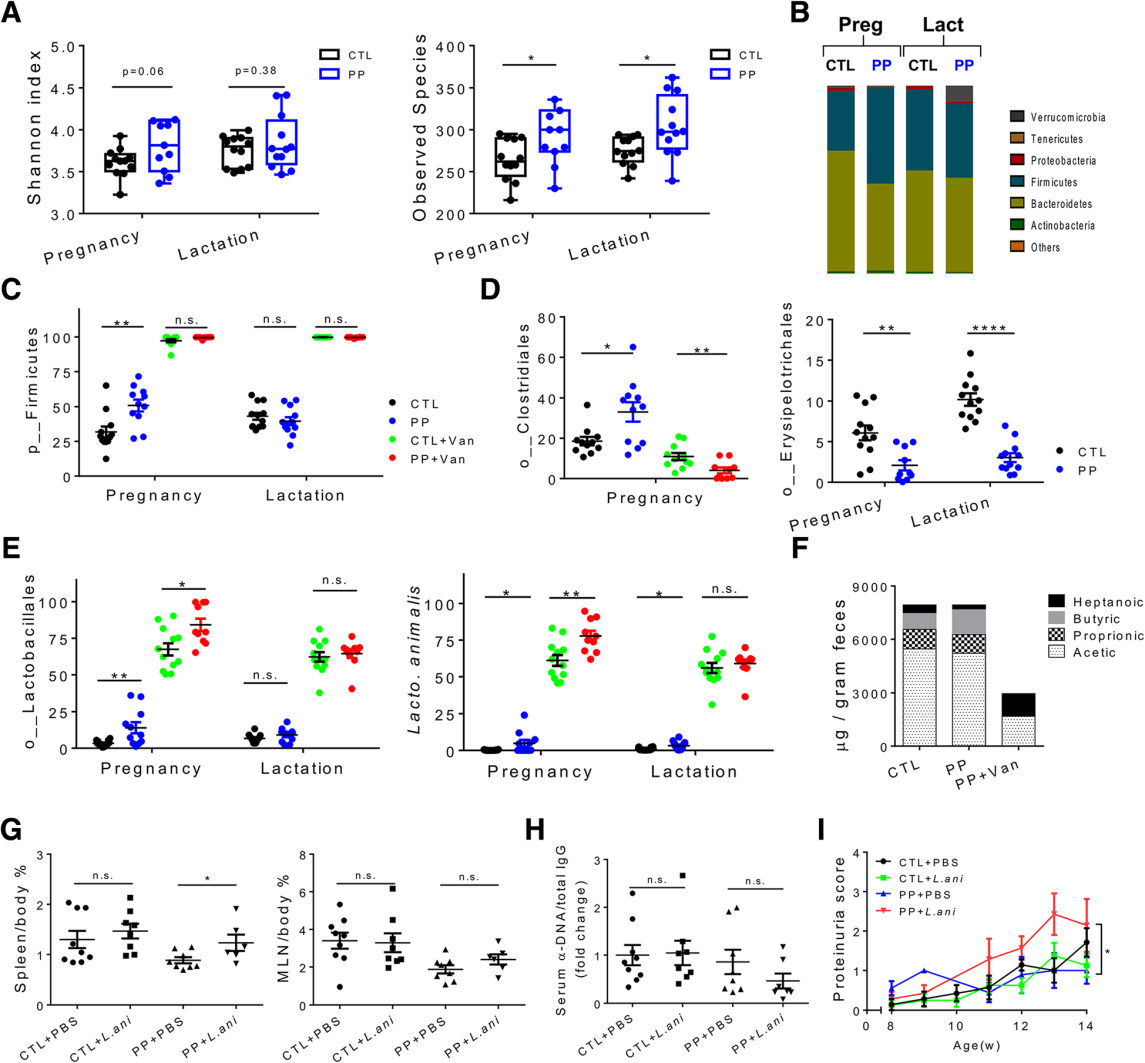
Identification of a bacterium responsible for the differential effects of vancomycin on control vs. PP MRL/lpr mice. Pregnancy: from 8 to 11 weeks of age. Lactation: from 12 to 14 weeks of age. **a** Shannon index and observed species of fecal microbiota at the 15 weeks of age (*n* = 12 per group). **b** Changes of fecal microbiota. Bacterial taxa at the phylum level are shown (*n* = 12 per group). **c** Relative abundance of Firmicutes in fecal microbiota of mice with or without vancomycin treatment (*n* ≥ 10 per group). **d** Relative abundance of Clostridiales and Erysipelotrichales in fecal microbiota (*n* ≥ 10 per group). **e** Relative abundance of Lactobacillales and *L. animalis* in fecal microbiota of mice with or without vancomycin treatment (*n* ≥ 10 per group). **f** Level of fecal SCFAs: butyric, proprionic, acetic, and heptanoic acids (*n* ≥ 6 per group). **g** Spleen and MLN to body weight ratios at 15 weeks of age (*n* ≥ 6 per group). **h** Ratio of anti-DNA IgG to total IgG in mouse serum at 15 weeks of age (*n* ≥ 7 per group). **i** Level of proteinuria over time (*n* ≥ 7 per group). *p < 0.05, **p < 0.01, ****p < 0.0001, n.s. not statistically significant

It is well established that pregnancy can influence gut microbiota community [29, 49]. However, the possible role of gut microbiota in the link between pregnancy and lupus flares remains uncovered. In MRL/lpr lupus-prone mice, we found that compared to age-matched control females, PP mice had modest increases of gut microbiota diversity and richness during pregnancy and lactation (Fig. 3a). The composition of gut microbiota changed as well (Fig. 3b). During pregnancy, at the phylum level, only Firmicutes was significantly increased (Fig. 3c). However, during lactation, Verrucomicrobia was greatly increased in the gut of PP mice (Additional file 1: Figure S3A). Under the phylum Firmicutes, Clostridiales and Lactobacillales significantly increased during pregnancy while Erysipelotrichales significantly decreased (Fig. 3d, e). In addition, at the order level, the relative abundance of Bacteroidales, Acholeplasmatales, Verrucomicrobiales, and Desulfovibrionales was all significantly changed during either pregnancy or lactation (Additional file 1: Figure S3C). Recently, a *Lachnospiraceae* species, *Ruminococcus gnavus*, has been implicated in lupus nephritis [50]. However, we did not observe statistically significant differences in either the *Lachnospiraceae* family or *R. gnavus*. While many bacterial species in the gut microbiota are able to produce SCFAs, primarily butyrate, acetate, and propionate [28], the variations of gut microbiota in control vs. PP mice did not influence the levels of SCFAs, although slightly more butyrate was noted in the feces of PP mice (Fig. 3f).

For mice receiving vancomycin, the changes of gut microbiota in control and PP mice were similar: Firmicutes, in particular Lactobacillales, flourished (Fig. 3c, e). Further analysis showed that one *Lactobacillus* species, *L. animalis*, accounted for the vast majority of Lactobacillales in vancomycin-treated mice (Fig. 3e and Additional file 1: Figure S3B). It is worth mentioning that the total bacterial load in the gut reduced more than 90% with vancomycin treatment (data not shown) As a result, the levels of 3 major SCFAs in the feces dramatically decreased (Fig. 3f). Conversely, the fecal heptanoic acid increased although the cause is unclear.

We decided to investigate the role of *L. animalis* as it dominated the gut microbiota of vancomycin-treated mice. While it belongs to a family with numerous species bearing probiotic properties, studies on *L. animalis* are limited and its influence on host biology is unknown. To test if the enriched *L. animalis* was responsible for the differential effects observed for vancomycin, we performed an experiment involving 4 groups: naïve mice gavaged with PBS (CTL + PBS) or *L. animalis* (CTL + *L.ani*), and PP mice gavaged with PBS (PP + PBS) or *L. animalis* (PP + *L.ani*). The weekly bacteria oral gavage was initiated from 9 weeks of age and lasted until 15 weeks of age, replacing vancomycin in the experimental design shown in Fig. 1a. Strikingly, the *L. animalis* gavage led to worsened splenomegaly in PP but not control mice (Fig. 3g). Neither the MLN weight nor the level of anti-DNA IgG in the circulation was changed (Fig. 3g, h), consistent with what was observed with vancomycin treatment (Fig. 1b, c). Furthermore, the bacteria gavage significantly worsened proteinuria only in PP mice (Fig. 3i). These results suggest that *L. animalis* recapitulated the effects of vancomycin and was able to exacerbate lupus nephritis in PP mice rather than naïve mice.

### Mechanisms behind the differential effects of vancomycin and *L. animalis* in control vs. PP MRL/lpr mice

#### Microbial translocation

Microbial translocation, caused by a leaky gut, has been recognized as an important factor affecting multiple autoimmune disorders including lupus [24, 51, 52]. In lupus-prone MRL/lpr mice, we found that a mixture of probiotics could reduce gut leakiness leading to attenuation of lupus nephritis [22]. In human lupus patients, the circulating endotoxin was highly elevated indicating a leaky gut [53]. Consistently, we found that in the serum of lupus patients, there were more antibodies against two major microbial antigens—lipopolysaccharide (LPS) and flagellin (Additional file 1: Figure S4A). This suggests increased entry of these antigens from the gut lumen and subsequent triggering of an immune response against them. The concentrations of these two antibodies significantly correlated with each other (Additional file 1: Figure S4B). As the patient samples were collected after disease onset, it remained unclear whether microbial translocation was a cause or consequence of lupus pathogenesis. We could partially answer this question in lupus-prone mice, as samples could be collected before disease onset. Thus, anti-LPS and anti-flagellin antibodies were examined in MRL/lpr mice at different disease stages as compared to age-matched MRL mice. MRL mice are control mice for MRL/lpr and do not develop disease during the time frame that MRL/lpr develop lupus [54]. We found that MRL/lpr mice at late disease stage (lpr-old, 15 weeks of age) had significantly higher IgG levels against the bacterial antigens than age-matched MRL mice (MRL-old) (Additional file 1: Figure S4C). Importantly, however, pre-disease MRL/lpr mice (lpr-young, 7 weeks of age) presented similar levels of these antibodies (Additional file 1: Figure S4C), suggesting that a leaky gut may have occurred prior to disease initiation. Like in human lupus, the levels of the two antibodies highly correlated with each other in lupus-prone mice (Additional file 1: Figure S4D). These results suggest that, rather than a consequence of disease, the leaky gut and subsequent microbial translocation are likely a causative factor in lupus pathogenesis.

In our previous study, vancomycin treatment enhanced barrier function and reduced microbial translocation in naïve MRL/lpr mice [21]. The decreased IgG against LPS confirmed that result (Additional file 1: Figure S4E). Gut microbiota is known as an important factor regulating intestinal barrier function [55]. However, despite the changes of gut microbiota during pregnancy and lactation (Fig. 3a–e), PP mice exhibited similar levels of antibodies against LPS as control mice (Additional file 1: Figure S4E). Vancomycin, possibly through decreasing the bacterial load, decreased the level of anti-LPS antibodies in both control and PP mice (Additional file 1: Figure S4E). This suggests that the exacerbation of lupus disease observed for vancomycin-treated PP mice was not due to microbial translocation. Moreover, while multiple *Lactobacillus* species have been described as enhancers of intestinal barrier function [22, 56, 57], *L. animalis* did not change the concentrations of anti-LPS antibodies in either naïve or PP mice (Additional file 1: Figure S4F). This further confirms that the differential response to vancomycin and *L. animalis* in control vs. PP mice cannot be explained by the changes of intestinal barrier function.

#### Regulation of Treg cells

**Fig. 4:**
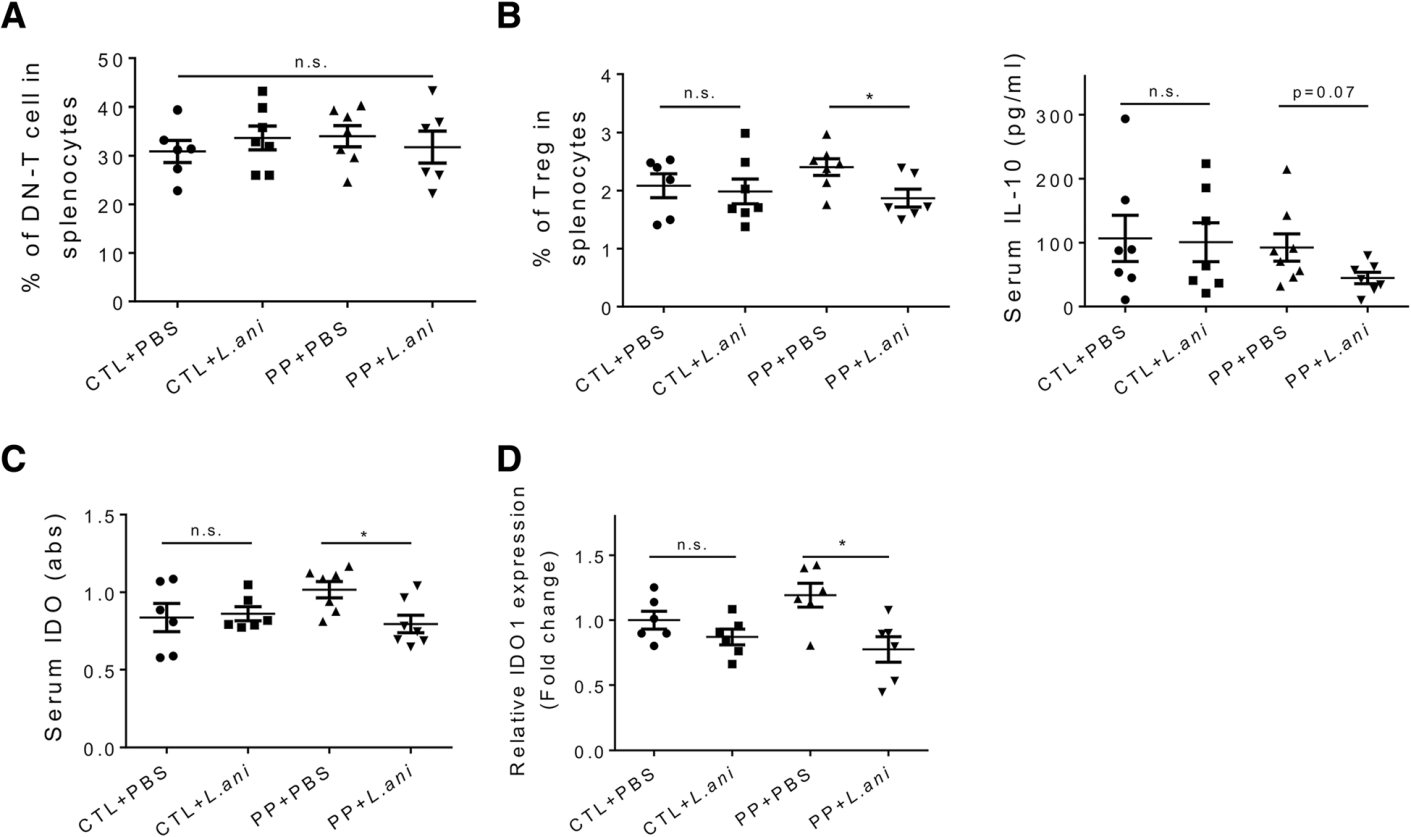
Regulation of Treg cells by *L. animalis* in PP MRL/lpr mice. **a** Percentage of DN-T cell in the spleen at 15 weeks of age. **b** Percentage of Treg cells in the spleen (left) and level of serum IL-10 (right) at 15 weeks of age. **c** Level of IDO in the mouse serum at 15 weeks of age. **d** Transcript level of IDO1 in the spleen at 15 weeks of age. In **a**–**d**, *n* ≥ 6 in each group. *p < 0.05, n.s. not statistically significant

Both vancomycin and *L. animalis* worsened lupus disease only in PP mice. However, unlike the effects of vancomycin on IL-17 producing cells (Fig. 2b–d), *L. animalis* gavages failed to modulate either DN-T cell (Fig. 4a) or Th17 cell (data not shown) responses in PP mice. We thus focused on the effects of *L. animalis* on splenic Treg cells and circulating anti-inflammatory IL-10 (Fig. 4b), where *L. animalis* gavages resembled the vancomycin treatment (Fig. 2e, f). More importantly, consistent with the effect of vancomycin (Fig. 2g), *L. animalis* significantly decreased serum IDO only in PP mice (Fig. 4c). Correspondingly, the bacterial gavage led to significantly lower IDO expression in the spleen (Fig. 4d), but not in the MLN (data not shown). These results suggest that *L. animalis* was able to inhibit IDO, similar to several other *Lactobacillus* species that have been reported to change IDO expression and activity [58–60]. Considering the dominant abundance of *L. animalis* upon vancomycin treatment, it is reasonable to believe that the enriched *L. animalis* was responsible for the vancomycin-driven IDO inhibition. As IDO can stimulate Treg cells during the development of autoimmunity [61], these results suggest that vancomycin and *L. animalis* downregulate Treg cells in PP mice through inhibiting IDO.

#### Involvement of IFNγ

**Fig. 5:**
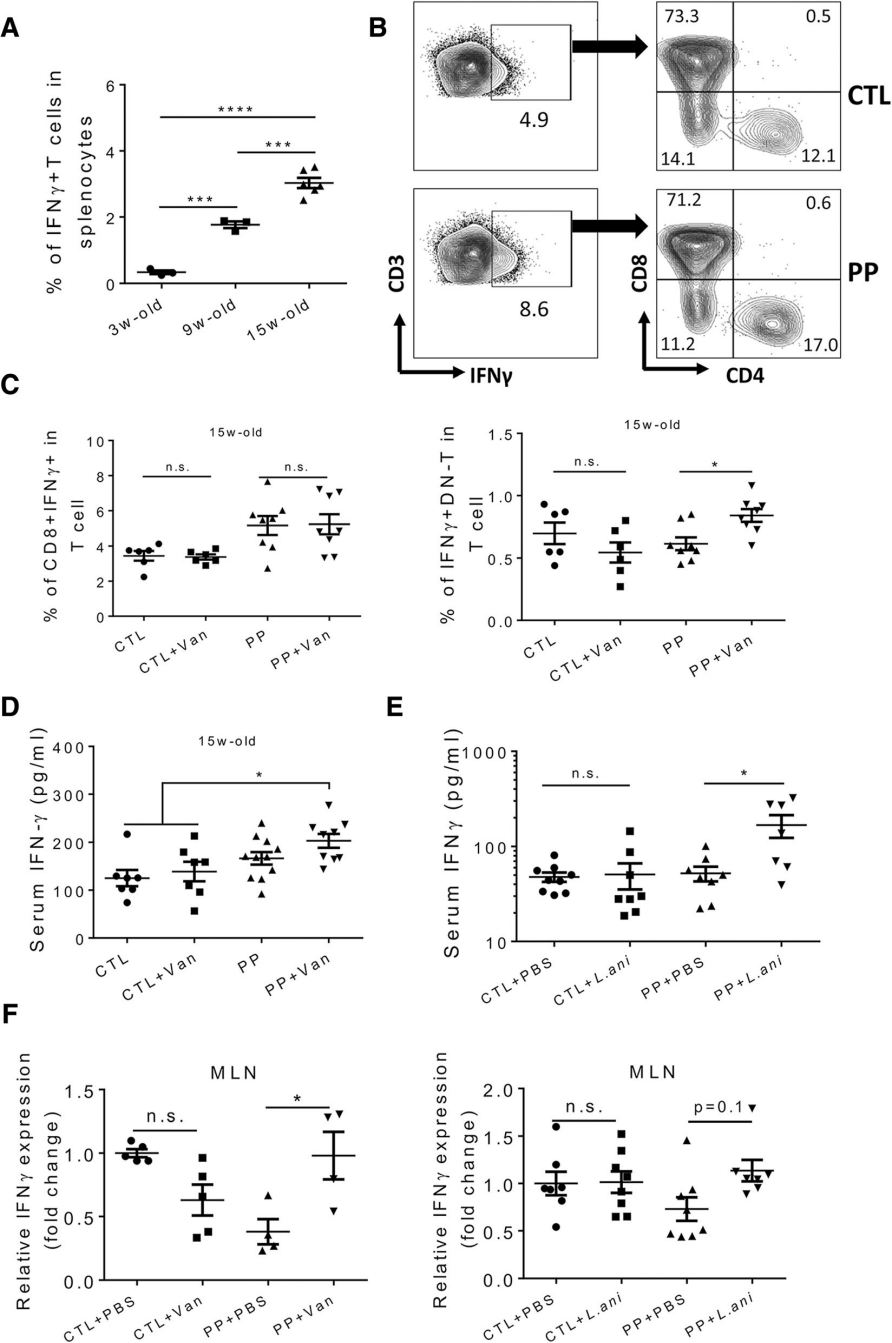
Enhancement of IFNγ production by both vancomycin and *L. animalis* in PP MRL/lpr mice. **a** Percentage of IFNγ producing T cells in the spleen of mice at different disease stages (*n* ≥ 3 per group). **b** FACS analysis of IFNγ producing T cells. **c** Percentage of CD8^+^IFNγ^+^ cells and CD4^-^CD8^-^IFNγ^+^ cells in splenic T cells at 15 weeks of age (*n* ≥ 6 per group). **d** Level of IFNγ in the mouse serum at 15 weeks of age (*n* ≥ 6 per group). **e** Level of IFNγ in the mouse serum at 15 weeks of age (*n* ≥ 7 per group). **f** Transcript level of IFNγ in the MLN. *p < 0.05, ***p < 0.001, ****p < 0.0001, n.s., not statistically significant

IFNγ is known as a pivotal proinflammatory cytokine that promotes lupus disease development [62]. In MRL/lpr mice, the percentage of IFNγ producing T cells in the spleen increased during the progression of disease (Fig. 5a). Notably, compared to IFNγ^+/+^ MRL/lpr mice, IFNγ^+/−^ MRL/lpr mice had an extended life span and ameliorated proteinuria, though autoantibody production was not affected [63]. In MRL/lpr mice that had experienced pregnancy and lactation, we found that the effects of vancomycin and *L. animalis* were mostly on lupus nephritis (Figs. 1e and 3i) but not autoantibody production (Figs. 1c and 3h), which coincides with the observation in IFNγ^+/−^ MRL/lpr mice. In addition, the strong correlation between IDO and IFNγ [64] suggests the need to investigate the possible involvement of IFNγ. We found that there were more T cells producing IFNγ in PP mice compared to control mice (Figs. 5b and 6c). Further analysis revealed that CD8^+^ T cells were the major producer of IFNγ in both types of mice (Fig. 5b). Interestingly, the proportion of CD8^+^IFNγ^+^ cells remained unchanged with or without vancomycin treatment in both control and PP mice (Fig. 5c). In contrast, vancomycin treatment significantly increased the ability of DN-T cells to produce IFNγ only in PP mice (Fig. 5c). The serum IFNγ level increased accordingly in this group of mice receiving vancomycin (Fig. 5d). Strikingly, oral *L. animalis* gavages recapitulated the effect of vancomycin in PP mice by increasing serum IFNγ (Fig. 5e). Similar results were obtained in the MLN (Fig. 5f). These results suggest that vancomycin and *L. animalis* exacerbated lupus nephritis through upregulating IFNγ.

Together, our results suggest that the different responses of naive vs. PP MRL/lpr mice to vancomycin or *L. animalis* were irrelevant to microbial translocation; instead, they were a consequence of differential regulation of Treg cells, IDO, and IFNγ.

## Discussion

We previously reported that in lupus-prone MRL/lpr mice, oral vancomycin treatment initiated after disease onset (9 weeks of age) attenuated lupus manifestations by decreasing splenomegaly and autoantibody production and ameliorating renal damage [21]. The beneficial effect was attributed to the ability of vancomycin to reduce IL-17 production from multiple cellular resources including DN-T and Th17 cells and to enhance intestinal barrier function and subsequently inhibiting the translocation of microbial antigens from the gut lumen into the circulation. In this study, our investigation started with a surprising observation that the same antibiotic treatment did not benefit mice which had experienced pregnancy and lactation. Instead, vancomycin significantly worsened splenomegaly and kidney damage in PP MRL/lpr mice, though anti-DNA IgG in the circulation was not changed. Unlike in naïve mice where vancomycin targeted IL-17 producing cells for downregulation [21], the same vancomycin treatment resulted in decreased Treg and B10 cells in PP mice. As a result, the production of anti-inflammatory IL-10 was hampered. At the same time, the production of proinflammatory IFNγ from DN-T cells increased in vancomycin-treated PP mice. Therefore, there was a shift of immune balance towards proinflammatory response that could explain how vancomycin exacerbated lupus disease in PP mice.

Vancomycin is not absorbed in the intestine [30]. Hence, the effect of oral vancomycin is limited in the gut lumen without causing systemic side effects. It is known that vancomycin can remove gram-positive bacteria from the gut but spare *Lactobacilli* [21, 31, 32]. In this study, vancomycin administration again enriched Lactobacillales. One *Lactobacillus* species, *L. animalis*, appeared to flourish in the gut microbiota. Isolated from alimentary canal of animals more than 3 decades ago [65], *L. animalis* has not drawn much attention and there is no evidence suggesting that this bacterium can colonize the human oral, gastrointestinal tract, or vagina [66]. In the *Lactobacillaceae* family, *L. animalis* was phylogenetically grouped into the *L. salivarius* clade [67]. A number of species in this clade have been reported to be able to interact with the mammalian host [68], but the information on *L. animalis* is very limited. In the present study, the enrichment of *L. animalis* in the vancomycin-treated gut prompted us to investigate its role in regulating lupus disease. We found that oral gavages of the bacterium did not affect naïve MRL/lpr mice, but exacerbated lupus nephritis in PP mice. Remarkably, the differential effects of *L. animalis* were highly consistent with the effects of vancomycin on naïve vs. PP mice, suggesting a possible role of *L. animalis* in mediating the action of vancomycin on lupus disease. For example, both vancomycin and *L. animalis* decreased Treg cells and IDO and increased IFNγ in PP mice. However, the effects of *L. animalis* and vancomycin were not identical. For instance, vancomycin improved the intestinal barrier function and reduced microbial translocation in both control and PP females; but *L. animalis* failed to exert such improvements. This is possibly due to the fact that vancomycin can remove many other bacteria in addition to enriching Lactobacilli.

While the diet and housing environment were the same between control and PP MRL/lpr mice, pregnancy, and lactation experiences resulted in significant changes of gut microbiota, affecting microbial diversity, species richness, and microbiota composition. Many gut bacteria are capable of producing SCFAs [28]. However, despite the observed microbiota changes, PP mice had similar levels of the 3 major SCFAs in the feces as naïve mice, suggesting that the differential effects that we have seen were not due to variations of SCFAs. It is widely accepted that lupus patients experiencing pregnancy bear an increased risk of lupus flares [11, 15]. Sex hormones are believed to trigger the flares, in spite of the tolerant immune response generated during pregnancy [47]. Here, we reported changes of gut microbiota after pregnancy and lactation in lupus-prone MRL/lpr mice, but whether these changes would contribute to the triggering of lupus flares requires further investigation. Nonetheless, given the known effects of gut microbiota on host hormones [27, 69], it is reasonable to speculate that gut microbiota variations during and after pregnancy may play a role in triggering lupus disease. Importantly, the gut microbiota—hormone axis may explain why in the current study, the changes of microbiota by vancomycin resulted in different immune responses and subsequently opposite disease outcomes in control vs. PP mice.

In PP mice, we found that oral vancomycin and particularly the enrichment of *L. animalis* reduced the percentage of Treg cells and lowered IL-10 production. Simultaneously, the production of IFNγ increased. Further analysis revealed that the elevated IDO during pregnancy was inhibited by *L. animalis*. This may explain how the regulatory immune response was dampened. IDO is known as a major factor that pregnant individuals rely on to generate immune tolerance for the fetus [48]. It is well established that IDO can induce Treg under normal condition and in various disorders [33, 34]. Notably, Treg cells, IL-10, and IDO are closely linked to IFNγ [64, 70]. Whether the increased IFNγ production observed in vancomycin-treated PP mice was due to a dampened regulatory response, or directly originated from the change of IDO, or both, remains unclear.

Interestingly, *L. animalis* is not the only *Lactobacillus* species that can modulate IDO expression. In simian immunodeficiency virus-infected macaques, the depletion of gut-resident *Lactobacillus* spp. was associated with increased IDO, and direct supplementation of *Lactobacillus*-containing probiotics significantly inhibited IDO [60]. In another report, oral feeding of *L. johnsonii* significantly reduced IDO and decreased the incidence of diabetes among BioBreeding diabetes-prone rats [59]. Mechanistically, it was suggested that bacteria-derived H_2_O_2_ from *L. johnsonii* was the cause for the decrease of IDO*.* On the contrary, feeding of live *L. reuteri* enhanced systemic IDO activity in mice [58].

The generation of autoantibodies against nuclear antigens such as DNA is a hallmark of lupus [8]. However, in the current study, the anti-DNA IgG titer was not changed in PP mice receiving vancomycin or *L. animalis* treatment. The lowered IL-10 and increased IFNγ may be the causes for the exacerbated manifestations in these mice. Coincidently, neither IL-10^+/−^ nor IFNγ^+/−^ MRL/lpr had altered autoantibody titers, while the loss of one allele resulted in worsened or attenuated lupus nephritis, respectively [71, 72]. These results suggest that IL-10 and IFNγ regulate lupus nephritis in an autoantibody-independent manner. Additionally, while autoantibodies can promote kidney damage, their importance for the pathogenesis of lupus nephritis is debatable. Indeed, T cells and other immune mediators may be more important in causing the tissue damage [73].

## Conclusions

Our study highlights the changes of gut microbiota structure during pregnancy and the differential effects of the same microbiota-modulating strategies (i.e., vancomycin or *L. animalis*) on disease manifestations in naïve vs. PP MRL/lpr mice. The enzyme IDO, which can be inhibited by specific *Lactobacillus* spp., appears to be the factor directing the differed immune responses and opposite disease outcomes. The ultimate goal of our research is to identify beneficial as well as pathogenic gut bacterial species and to develop therapeutic strategies that are able to modulate the gut microbiota community towards a beneficial effect. For patients with autoimmune lupus, diet and probiotics are the two relatively easy and acceptable approaches that can potentially improve disease management through modulating the gut microbiota [22, 74]. However, it is challenging to achieve this goal due to the complexity of the disease pathologies, the complexity of gut microbiota, and the differences of gut microbiota communities among individuals. In future investigations, we plan to focus on lupus nephritis, the leading cause of mortality in lupus patients, to further delineate the role of gut microbiota in the link between pregnancy and exacerbated lupus.

## Methods

### Mice and vancomycin treatment

MRL/MpJ (MRL, stock number 000486) and MRL/MpJ-*Fas*^*lpr*^/J (MRL/lpr, stock number 000485) mice were purchased from The Jackson Laboratory (Bar Harbor, ME) and bred and maintained in a specific pathogen-free facility according to the requirements of the Institutional Animal Care and Use Committee (IACUC) at Virginia Tech (Animal Welfare Assurance Number: A3208-01). CO_2_ was used for euthanasia according to the IACUC protocol. All experiments were performed in accordance with relevant guidelines and regulations. To generate PP mice, 8-week-old female MRL/lpr mice were mated with age-matched male mice. Only the mice delivered at 11 weeks old (± 1 day) were used in the study. Vancomycin (2 g/L) was given in the drinking water from 9 weeks old till 15 weeks of age (endpoint). The drinking water containing vancomycin was replenished once a week.

### Bacteria culture and gavage

*Lactobacillus animalis* (35046) was purchased from ATCC (Manassas, VA) and cultured in Lactobacilli MRS Broth (BD Biosciences) according to the suggested culture method by ATCC. *L. animalis* was freshly cultured every week and orally gavaged, once a week, to MRL/lpr mice from 9 weeks of age until dissection at 15 weeks.

### Microbiota sampling and analysis

Fecal microbiota samples were obtained by taking the mouse out of the cage and collecting a fecal pellet. Samples were stored at − 80 °C till being processed at the same time. Sample homogenization, cell lysis, and DNA extraction were performed as previously described [19]. PCR were performed, and purified amplicons were sequenced bidirectionally on an Illumina MiSeq at Argonne National Laboratory. 16S rRNA sequencing data were analyzed as described previously [19].

### Gas chromatography measurement of SCFAs

Fecal samples were acidified using phosphoric acid immediately before analysis. Injector settings: temperature 200 °C; carrier, hydrogen; injection mode, split (ratio 2:1). Temperature program: initial temperature of 80 °C held for 3 min and then increase temperature at a rate of 6 °C per minute to 140 °C and hold for 1 min. The Flame Ionization Detector Settings: temperature, 250 °C; hydrogen flow, 35 ml/minute; air flow, 350 ml/minute; makeup flow (nitrogen), 15 ml/minute; and total makeup (makeup + column flow), 30 ml/minute.

### ELISA

Mouse sera separated after blood clotting were saved at − 20 °C until use. Human plasma samples were purchased from AllCells (Alameda, CA). For measuring anti-DNA IgG, anti-LPS IgG, and anti-flagellin IgG, high-binding plates were coated overnight at 4 °C with 100 μl of calf thymus DNA (100 μg/ml, Invitrogen), *Escherichia coli* LPS (10 μg/ml, Sigma), or *Salmonella* flagellin (1 μg/ml, Enzo Life Sciences), respectively. After sequential incubations on the second day with the samples and secondary antibodies (anti-human IgG-HRP or anti-mouse IgG-HRP, Invitrogen), TMB substrate solution (Thermo Fisher Scientific) was added, followed by the stop solution. Plates were read at 450 nm absorbance with the SpectraMax M5 microplate reader (Molecular Devices LLC). Mouse serum total IgG was determined with mouse IgG kit (Bethyl Laboratories). Cytokine and enzyme concentrations were determined with mouse IL-10, IL-6, IFNγ (Biolegend), IL-35 (LifeSpan BioSciences), and IDO (MyBioSource) ELISA kits, respectively, according to the manufacturers’ instructions.

### Renal function

Urine was collected weekly starting from disease onset at 9 weeks of age, and all samples were stored at − 20 °C till analyzed at the same time with a Pierce Coomassie Protein Assay Kit (Thermo Scientific). Proteinuria level was scored as: 0 (0–50 mg/dl), 1 (50–100 mg/dl), 2 (100–200 mg/dl), 3 (200–400 mg/dl), and 4 (> 400 mg/dl). When mice were euthanized at 15 weeks of age, the kidney was fixed in formalin for 24 h, paraffin embedded, sectioned, and stained with periodic acid-Schiff (PAS) at the Histopathology Laboratory at Virginia-Maryland College of Veterinary Medicine. Slides were read with an Olympus BX43 microscope. All the slides were scored in a blinded fashion by a certified veterinary pathologist. Glomerular lesions were graded on a scale of 0 to 3 for each of the following 5 categories: increased cellularity, increased mesangial matrix, necrosis, the percentage of sclerotic glomeruli, and the presence of crescents. Tubulointerstitial lesions were graded on a scale of 0 to 3 for each of the following four categories: presence of peritubular mononuclear infiltrates, tubular damage, interstitial fibrosis, and vasculitis.

### Cell isolation and flow cytometry

Spleens were collected and mashed in 70-μm cell strainers with complete media. Red blood cells were lysed with RBC lysis buffer (eBioscience). For surface staining, cells were blocked with anti-mouse CD16/32 (eBioscience), stained with fluorochrome-conjugated antibodies, and analyzed with BD FACSAria II flow cytometer (BD Biosciences, San Jose, CA). For intracellular staining, Foxp3 Fixation/Permeabilization kit (eBioscience) was used. Anti-mouse antibodies used in this study include the following: CD3-APC, CD4-PE-Cy7, CD8-FITC, IL-10-PerCP-Cy5.5, IFNγ-APC-Cy7, IL-17A-PE, Foxp3-PE, and CD19-APC (Biolegend) and RORγt-BV421 (BD Biosciences). Flow cytometry data were analyzed with FlowJo.

### RT-qPCR

Spleens were homogenized with Bullet Blender homogenizer (Next Advance), and total RNA was extracted with RNeasy Plus Mini Kit (Qiagen) according to the manufacturers’ instructions. Genomic DNA was removed by digestion with RNasefree DNase I (Qiagen). Reverse transcription (RT) was performed by using iScript cDNA Synthesis Kit (Bio-Rad). Quantitative PCR (qPCR) was performed with iTaq Universal SYBR Green Supermix (Bio-Rad) and ABI 7500 Fast Real-Time PCR System (Applied Biosystems). Relative quantities were calculated using L32 as the housekeeping gene. Primer sequences for mouse *L32* and *IDO1* are available upon request.

### Statistical analysis

For the comparison of two groups, unpaired Student’s *t* test was used. For the comparison of more than two groups, one-way ANOVA and Tukey’s post-test were used. Results were considered statistically significant when *p* < 0.05. All analyses were performed with Prism GraphPad.

## Additional file

Additional file 1: Supplementary **Figures S1–S4.** (PDF 380 kb)

## Data Availability

The datasets generated and analyzed during the current study are available in the NCBI SRA accession number SRP182989.

## Abbreviations

B10: IL-10 producing B cell
DN-T: Double negative T cells
IDO: Indoleamine 2,3-dioxygenase
*L. animalis*: *Lactobacillus animalis*
LPS: Lipopolysaccharide
MLN: Mesenteric lymph node
SCFAs: Short chain fatty acids
Th17: T-helper 17 cells
Treg: Regulatory T cell

## References

[1] Davidson A, Diamond B (2001). Autoimmune diseases. N Engl J Med.

[2] Cooper GS, Bynum MLK, Somers EC (2009). Recent insights in the epidemiology of autoimmune diseases: improved prevalence estimates and understanding of clustering of diseases. Journal of Autoimmunity.

[3] Khashan AS (2011). Pregnancy and the risk of autoimmune disease. Plos One.

[4] Waldorf KMA, Nelson JL (2008). Autoimmune disease during pregnancy and the microchimerism legacy of pregnancy. Immunological investigations.

[5] Kumar P, Magon N (2012). *Hormones in pregnancy*. Niger Med J.

[6] Tincani A (2016). Pregnancy in patients with autoimmune disease: a reality in 2016. Autoimmunity Reviews.

[7] Borchers AT (2010). The implications of autoimmunity and pregnancy. Journal of Autoimmunity.

[8] Tsokos GC (2011). Systemic lupus erythematosus. N Engl J Med.

[9] Kiriakidou M (2013). Systemic lupus erythematosus. Ann Intern Med.

[10] Almaani S, Meara A, Rovin BH (2017). Update on lupus nephritis. Clin J Am Soc Nephrol.

[11] Eudy AM (2018). Effect of pregnancy on disease flares in patients with systemic lupus erythematosus. Annals of the Rheumatic Diseases.

[12] Carp HJA, Selmi C, Shoenfeld Y (2012). The autoimmune bases of infertility and pregnancy loss. Journal of Autoimmunity.

[13] Chen JS (2015). Pregnancy outcomes in women with rare autoimmune diseases. Arthritis & Rheumatology.

[14] Moroni G (2016). Maternal outcome in pregnant women with lupus nephritis. A prospective multicenter study. J Autoimmun.

[15] McDonald EG (2018). Monitoring of systemic lupus erythematosus pregnancies: a systematic literature review. Journal of Rheumatology.

[16] Kåhrström Christina Tobin, Pariente Nonia, Weiss Ursula (2016). Intestinal microbiota in health and disease. Nature.

[17] Mu QH, Zhang HS, Luo XM (2015). SLE: Another autoimmune disorder influenced by microbes and diet?. Frontiers in Immunology.

[18] Hevia, A., et al., Intestinal dysbiosis associated with systemic lupus erythematosus. Mbio, 2014. 5(5). 10.1128/mBio.01548-14 PMC4196225 25271284

[19] Zhang HS (2014). Dynamics of gut microbiota in autoimmune lupus. Applied and Environmental Microbiology.

[20] Luo XM (2018). Gut microbiota in human systemic lupus erythematosus and a mouse model of lupus. Applied and Environmental Microbiology.

[21] Mu Q (2017). Antibiotics ameliorate lupus-like symptoms in mice. Sci Rep.

[22] Mu Q (2017). Control of lupus nephritis by changes of gut microbiota. Microbiome.

[23] Vieira SM (2018). Translocation of a gut pathobiont drives autoimmunity in mice and humans. Science.

[24] Mu Q (2017). Leaky gut as a danger signal for autoimmune diseases. Front Immunol.

[25] Zegarra-Ruiz DF, et al. A diet-sensitive commensal Lactobacillus strain mediates TLR7-dependent systemic autoimmunity. Cell Host Microbe. 2018. 10.1016/j.chom.2018.11.009 PMC6377154 30581114

[26] Greiling TM (2018). Commensal orthologs of the human autoantigen Ro60 as triggers of autoimmunity in lupus. Science Translational Medicine.

[27] Li JY (2016). Sex steroid deficiency-associated bone loss is microbiota dependent and prevented by probiotics. Journal of Clinical Investigation.

[28] Morrison DJ, Preston T (2016). Formation of short chain fatty acids by the gut microbiota and their impact on human metabolism. Gut Microbes.

[29] Nuriel-Ohayon M, Neuman H, Koren O. Microbial changes during pregnancy, birth, and infancy. Frontiers in Microbiology. 2016;7. 10.3389/fmicb.2016.01031 PMC4943946 27471494

[30] Rao S (2011). Systemic absorption of oral vancomycin in patients with Clostridium difficile infection. Scand J Infect Dis.

[31] Robinson CJ, Young VB (2010). Antibiotic administration alters the community structure of the gastrointestinal micobiota. Gut Microbes.

[32] Murphy EF (2013). Divergent metabolic outcomes arising from targeted manipulation of the gut microbiota in diet-induced obesity. Gut.

[33] Baban B (2009). IDO activates regulatory T cells and blocks their conversion into Th17-Like T cells. Journal of Immunology.

[34] Sharma MD (2009). Indoleamine 2,3-dioxygenase controls conversion of Foxp3(+) Tregs to TH17-like cells in tumor-draining lymph nodes. Blood.

[35] Munn DH (2011). Indoleamine 2,3-dioxygenase, Tregs and cancer. Current Medicinal Chemistry.

[36] Isenberg DA (2007). Fifty years of anti-ds DNA antibodies: are we approaching journey’s end?. Rheumatology (Oxford).

[37] Kimura A, Kishimoto T (2010). IL-6: regulator of Treg/Th17 balance. Eur J Immunol.

[38] Tackey E, Lipsky PE, Illei GG (2004). Rationale for interleukin-6 blockade in systemic lupus erythematosus. Lupus.

[39] Crispin JC (2008). Expanded double negative T cells in patients with systemic lupus erythematosus produce IL-17 and infiltrate the kidneys. J Immunol.

[40] Martina MN (2015). Double negative (DN) alphabeta T cells: misperception and overdue recognition. Immunol Cell Biol.

[41] Korn T (2009). IL-17 and Th17 cells. Annu Rev Immunol.

[42] Arellano B, Graber DJ, Sentman CL (2016). Regulatory T cell-based therapies for autoimmunity. Discov Med.

[43] Eisenstein EM, Williams CB (2009). The T(reg)/Th17 cell balance: a new paradigm for autoimmunity. Pediatr Res.

[44] Mauri C, Menon M (2017). Human regulatory B cells in health and disease: therapeutic potential. J Clin Invest.

[45] Choi J (2015). IL-35 and autoimmunity: a comprehensive perspective. Clin Rev Allergy Immunol.

[46] Lee CC (2018). Macrophage-secreted interleukin-35 regulates cancer cell plasticity to facilitate metastatic colonization. Nat Commun.

[47] Guleria I, Sayegh MH (2007). Maternal acceptance of the fetus: true human tolerance. J Immunol.

[48] Kudo Y (2013). The role of placental indoleamine 2,3-dioxygenase in human pregnancy. Obstet Gynecol Sci.

[49] Koren O (2012). Host remodeling of the gut microbiome and metabolic changes during pregnancy. Cell.

[50] Azzouz D, et al. Lupus nephritis is linked to disease-activity associated expansions and immunity to a gut commensal. Ann Rheum Dis. 2019. 10.1136/annrheumdis-2018-214856 PMC6585303 30782585

[51] Fasano A (2012). Leaky gut and autoimmune diseases. Clin Rev Allergy Immunol.

[52] Manfredo Vieira S (2018). Translocation of a gut pathobiont drives autoimmunity in mice and humans. Science.

[53] Shi L (2014). The SLE transcriptome exhibits evidence of chronic endotoxin exposure and has widespread dysregulation of non-coding and coding RNAs. PLoS One.

[54] Richard ML, Gilkeson G (2018). Mouse models of lupus: what they tell us and what they don’t. Lupus Science & Medicine.

[55] Kelly JR (2015). Breaking down the barriers: the gut microbiome, intestinal permeability and stress-related psychiatric disorders. Front Cell Neurosci.

[56] Eun CS (2011). Lactobacillus casei prevents impaired barrier function in intestinal epithelial cells. APMIS.

[57] Sultana R, McBain AJ, O’Neill CA (2013). Strain-dependent augmentation of tight-junction barrier function in human primary epidermal keratinocytes by Lactobacillus and Bifidobacterium lysates. Appl Environ Microbiol.

[58] Forsythe P, Inman MD, Bienenstock J (2007). Oral treatment with live Lactobacillus reuteri inhibits the allergic airway response in mice. Am J Respir Crit Care Med.

[59] Valladares R (2013). Lactobacillus johnsonii inhibits indoleamine 2,3-dioxygenase and alters tryptophan metabolite levels in BioBreeding rats. FASEB J.

[60] Vujkovic-Cvijin I (2015). Gut-resident Lactobacillus abundance associates with IDO1 inhibition and Th17 dynamics in SIV-infected macaques. Cell Rep.

[61] Lippens C (2016). IDO-orchestrated crosstalk between pDCs and Tregs inhibits autoimmunity. Journal of Autoimmunity.

[62] Pollard KM (2013). Interferon-gamma and systemic autoimmunity. Discov Med.

[63] Balomenos D, Rumold R, Theofilopoulos AN (1998). Interferon-gamma is required for lupus-like disease and lymphoaccumulation in MRL-lpr mice. J Clin Invest.

[64] Heyes MP (1991). Relationship between interferon-gamma, indoleamine-2,3-dioxygenase and tryptophan. FASEB J.

[65] Dent VE, Williams RAD (1982). Lactobacillus-animalis sp-nov, a new species of Lactobacillus from the alimentary canal of animals. Zentralblatt Fur Bakteriologie Mikrobiologie Und Hygiene I Abteilung Originale C-Allgemeine Angewandte Und Okologische Mikrobiologie.

[66] O’Callaghan J, O’Toole PW (2013). Lactobacillus: host-microbe relationships. Between Pathogenicity and Commensalism.

[67] Zhang ZG (2011). Phylogenomic reconstruction of lactic acid bacteria: an update. Bmc Evolutionary Biology.

[68] Neville BA, O’Toole PW (2010). Probiotic properties of Lactobacillus salivarius and closely related Lactobacillus species. Future Microbiology.

[69] Kwa M, et al. The intestinal microbiome and estrogen receptor-positive female breast cancer. Jnci-Journal of the National Cancer Institute. 2016;108(8). 10.1093/jnci/djw029 PMC5017946 27107051

[70] Panduro M, Benoist C, Mathis D (2018). T-reg cells limit IFN-gamma production to control macrophage accrual and phenotype during skeletal muscle regeneration. Proceedings of the National Academy of Sciences of the United States of America.

[71] Yin ZN (2002). IL-10 regulates murine lupus. Journal of Immunology.

[72] Balomenos D, Rumold R, Theofilopoulos AN (1998). Interferon-gamma is required for lupus-like disease and lymphoaccumulation in MRL-lpr mice. Journal of Clinical Investigation.

[73] Suarez-Fueyo A, Bradley SJ, Tsokos GC (2016). T cells in systemic lupus erythematosus. Current Opinion in Immunology.

[74] Vieira SM, Pagovich OE, Kriegel MA (2014). Diet, microbiota and autoimmune diseases. Lupus.

