# Supplementary material for: Pregnancy and lactation interfere with the response of autoimmunity to modulation of gut microbiota
Source: Microbiome. 2019 Jul 16;7:105. doi: 10.1186/s40168-019-0720-8 (PMC6635999; doi:10.1186/s40168-019-0720-8)
Supplement: Supplementary file 1 — Supplementary Figures S1–S4. (PDF 380 kb) [file 40168_2019_720_MOESM1_ESM.pdf]

Supplementary Figure 1.

A **B**

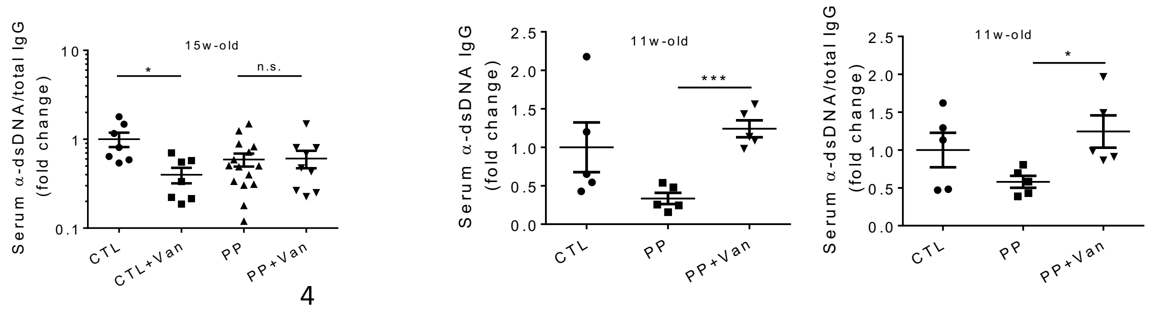

(A) Ratio of anti-dsDNA IgG to total IgG in the mouse serum at 15 weeks of age (n≥7 per group). (B) Level of anti-dsDNA IgG in the mouse serum and its ratio to total IgG at 11 weeks of age (n≥5 per group). **p*<0.05, ****p*<0.001, n.s.: not statistically significant.

Supplementary Figure 2.

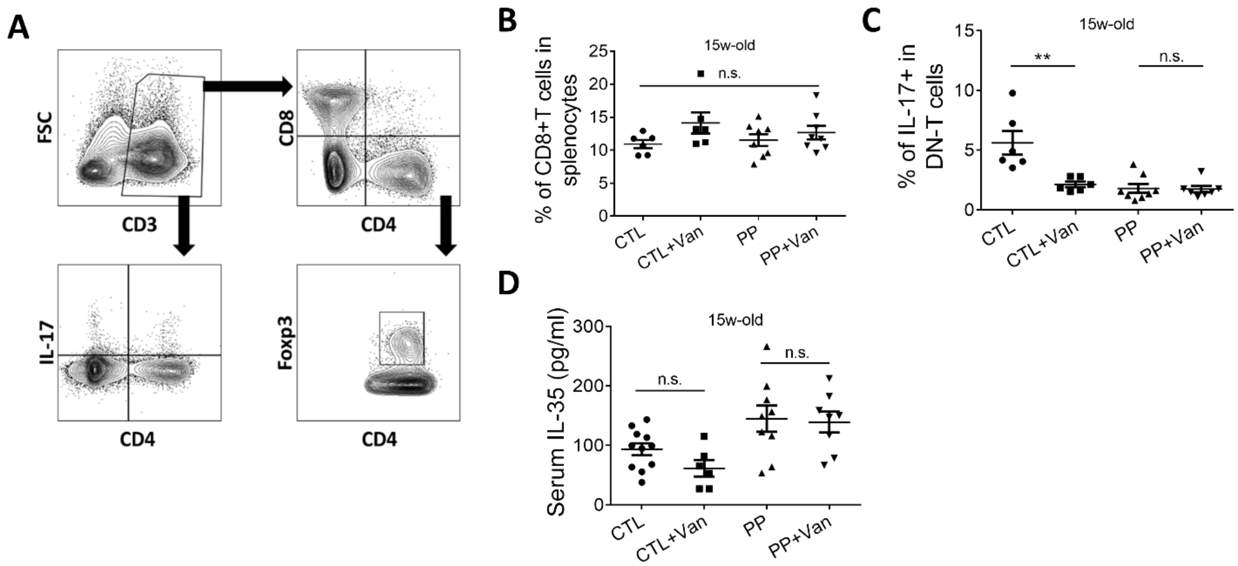

(A) Gating strategies for IL-17^{+} producing T cells and Treg cells. (B) Percentage of $CD8^{+}$ T cells ($CD3^{+}CD4^{-}CD8^{+}$) in the spleen at 15 weeks of age (n≥6 per group). (C) Percentage of IL-17 producing cells in splenic DN-T cells at 15 weeks of age (n≥6 per group). (D) Level of IL-35 in the mouse serum at 15 weeks of age (n≥6 per group). ***p*< 0.01, n.s.: not statistically significant.

Supplementary Figure 3

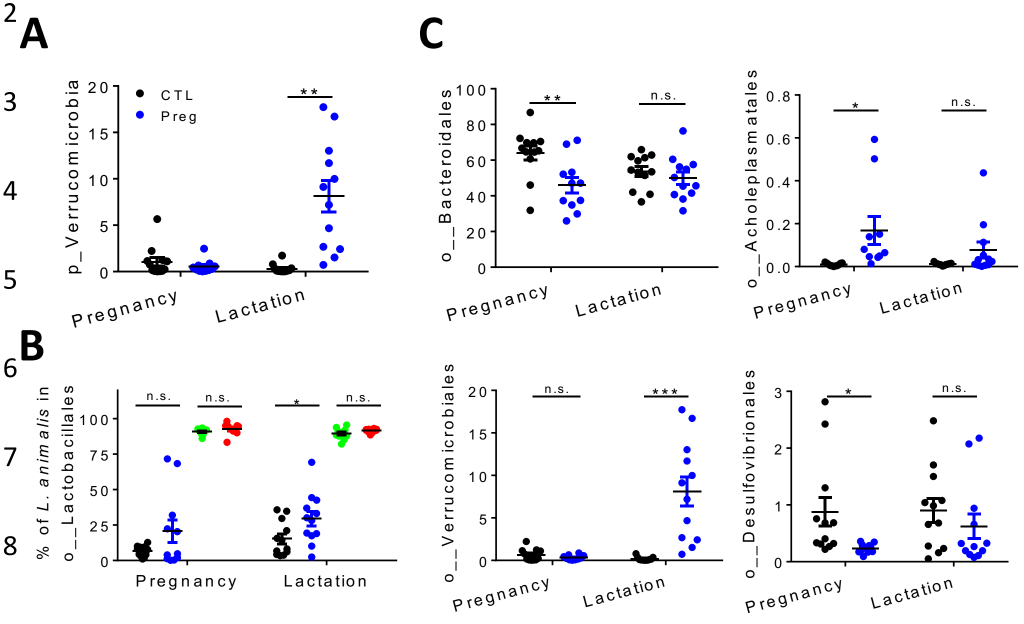

(A) Relative abundance of Verrucomicrobia in fecal microbiota (n=12 per group). (B) Percentage of *L. animalis* in Lactobacillales in the feces of mice at different ages with or without vancomycin treatment (n≥10 per group). (C) Relative abundance of Bacteroidales, Acholeplasmatales, Verrucomicrobiales and Desulfovibrionales in fecal microbiota at different ages (n=12 per group). *p<0.05, **p<0.01, ***p<0.001, n.s.: not statistically significant.

Supplementary Figure 4

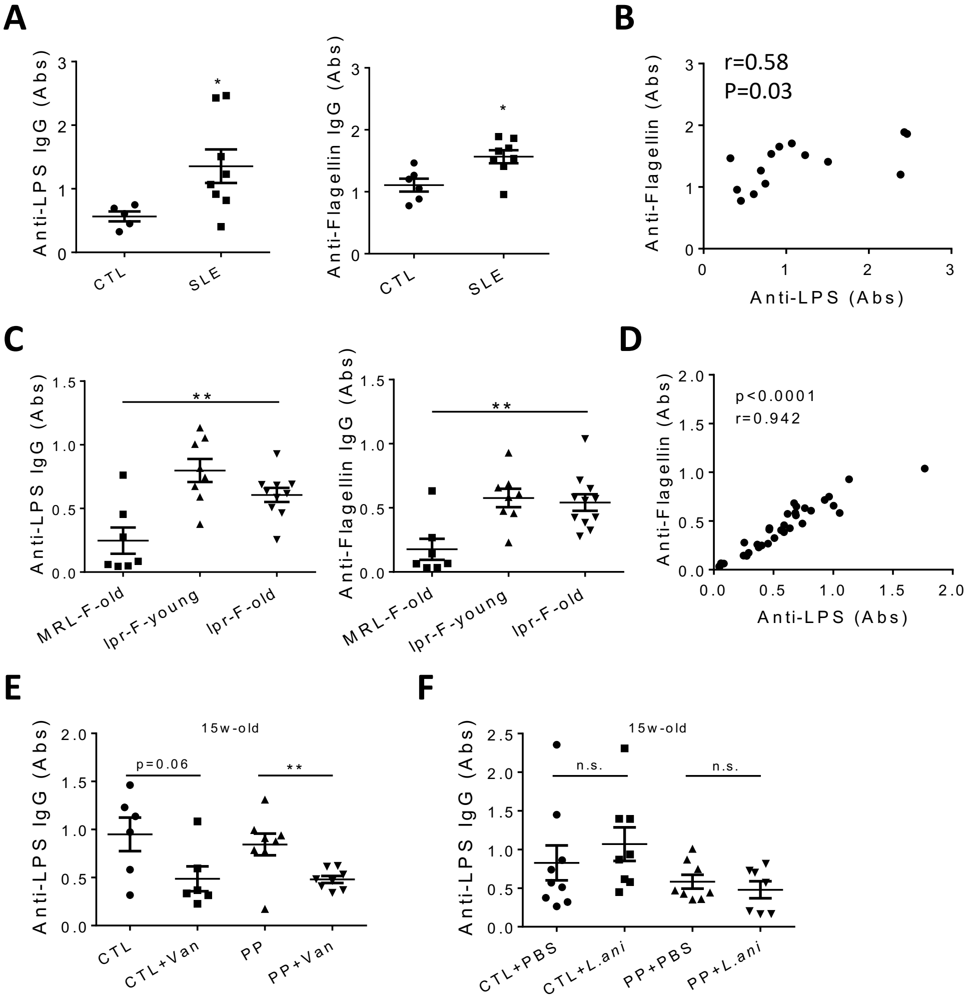

(A) Level of anti-LPS and anti-flagellin IgG in the plasma of human lupus patients and healthy controls (n≥5 per group). (B) Correlation analysis of human anti-LPS IgG and anti-flagellin IgG. (C) Level of anti-LPS and anti-flagellin IgG in the mouse serum. lpr-F-old: female MRL/lpr mice at 15 weeks of age. lpr-F-young: female MRL/lpr mice at 7 weeks of age. MRL-F-old: female MRL mice at 15 weeks of age (n≥7 per group). (D) Correlation analysis of mouse anti-LPS IgG and anti-flagellin IgG. (E) Level of anti-LPS IgG in mouse serum at 15 weeks of age (n≥6 per group). (F) Level of anti-LPS IgG in mouse serum at 15 weeks of age (n≥7 per group). **p*<0.05, ***p*<0.01, n.s.: not statistically significant.
